# Preferences and challenges regarding medical decision-making among patients with a migration background in Belgium: a focus group study

**DOI:** 10.1186/s13690-025-01648-7

**Published:** 2025-07-01

**Authors:** Amina Yakhlaf, Flore Vermijs, Katrien Bombeke, Veerle Buffel, Sarah Van de Velde, Josefien van Olmen, Paul Van Royen, Edwin Wouters, Nina Van Eekert

**Affiliations:** 1 Centre for Population, Family and Health, Department of Sociology, University of Antwerp, Sint-Jacobstraat 2-4, Antwerp, 2000 Belgium; 2 Primary and Interdisciplinary Care Antwerp, Department of Family Medicine and Population Health, University of Antwerp, Doornstraat 331, Wilrijk, Antwerp, 2610 Belgium; 3 Brussels Institute for Social and Population Studies, Department of Sociology, Vrije Universiteit Brussel, Pleinlaan 5, Brussels, 1050 Belgium; 4 Institute for Family and Sexuality Studies & Sociology Department, KU Leuven, ON5 Herestraat 49 - bus 1038, Louvain, Belgium

**Keywords:** Medical decision-making, Patients with migration background, Family involvement, Information exchange, Focus group discussions

## Abstract

**Background:**

The growing cultural diversity in Belgium, with over one third of the population having a migration background, presents distinct challenges for primary healthcare, particularly in doctor-patient interactions. Medical decision-making (MDM) is at the core of clinical practice in primary healthcare. According to existing literature, MDM may be complicated not only by general challenges arising in intercultural health communication, but also by specific cultural preferences concerning patient autonomy. This study aims to examine preferences and challenges regarding MDM among patients with a migration background in Belgium, from the perspectives of patients and general practitioners (GPs).

**Methods:**

Data were collected through five focus group discussions (FGDs), organised in November and December 2023. Three FGDs involved patients from the major migrant population groups in Belgium (Moroccan (*n* = 6), Turkish (*n* = 6), and sub-Saharan African (*n* = 7), and two FGDs involved GPs (*n* = 13). The data were analysed through reflexive thematic analysis.

**Results:**

Our results show that preferences and challenges revolved around four key components of MDM: Exchange of medical information, decision-making agency, patient-provider relationship, and treatment plan. These preferences and challenges were shaped by a complex interplay of factors at the individual (e.g., patient education, provider attitudes), interpersonal (e.g., family dynamics, language barriers), institutional (e.g., legal framework, medical education), and cultural levels (e.g., religion, health beliefs).

**Conclusions:**

Our study highlights the importance of adopting an intersectional and multilayered perspective on MDM, which considers how various factors interact and shape preferences and challenges in MDM, depending on the individual and their context. Although it is impossible for GPs to be aware of every cultural preference, this study shows how GPs can engage in more culturally sensitive interactions with their patients. By also addressing the institutional factors that contribute to challenges in MDM, this approach can improve person-centred care by better accommodating each patient’s unique needs and preferences.

**Supplementary Information:**

The online version contains supplementary material available at 10.1186/s13690-025-01648-7.

**Table:** 

Text box 1. Contributions to the literature
– Little is known about preferences and challenges regarding medical decision-making (MDM) among patients with a migration background in Belgium.
– This interdisciplinary study, integrating insights from sociology and medicine, represents the first qualitative research on this topic, exploring perspectives from both patients and general practitioners.
– Our findings show that preferences and challenges can be categorised into four components of MDM: Exchange of medical information, decision-making agency, patient-provider relationship, and treatment plan.
– These preferences and challenges are shaped by a myriad of factors, underscoring the importance of adopting an intersectional and multilayered perspective on MDM.

## Background

Due to several waves of immigration, over one-third of the Belgian population now has a migration background. A person with a migration background is defined as an individual who either has a non-Belgian nationality at birth, or has at least one parent with a non-Belgian nationality at birth [1]. Individuals with a Turkish, Moroccan and sub-Saharan African migration background constitute the largest non-European migrant groups in Belgium, due to various historical factors. These include bilateral agreements that attracted labour migrants from Morocco and Turkey in the 1960s, Belgium’s colonial history in Congo and Rwanda [2], and subsequent waves of family reunification [3]. Cultural diversity introduces distinct challenges in primary healthcare. Despite a general high patient satisfaction with Belgium’s healthcare system, significant challenges remain in ensuring equitable healthcare for vulnerable populations, particularly those with a migration background [4, 5].

As the primary point of contact, general practitioners (GPs) are directly confronted with challenges in consultations with patients with a migration background [6, 7], particularly during medical decision-making (MDM). MDM is central to clinical practice. It encompasses both low- and high-stakes decisions, including treatment options, hospital referrals, diagnostic examinations, and disease prevention and management. MDM is a complex and collaborative process involving healthcare providers and patients working together to make decisions while considering both their values and preferences [8]. Effective communication between patients and GPs is vital for MDM [9]. It promotes mutual understanding and encourages positive interactions, leading to optimal outcomes. Challenges such as limited language proficiency, cultural differences, and unfamiliarity with the healthcare system, can complicate MDM, particularly with patients from non-European backgrounds [10–12].

A shared model of MDM, often referred to as shared decision-making (SDM) has gained significant importance in recent decades. Unlike traditional MDM models, where healthcare providers often control the decision-making process, SDM places the patient at the centre. The SDM model encourages patients to actively participate in discussions and make final decisions together with their GP [13, 14]. In current primary care practice in Belgium, MDM ideally aligns with an SDM model, which reflects the growing emphasis on patients’ individual self-determination [15]. However, in many non-European cultures, illness is often seen as a family matter [16]. Collectivist ideals, deeply rooted in Moroccan, Turkish, and sub-Saharan African cultures, shape MDM by prioritising family-centred decision-making over individual patient autonomy [15]. This can create tensions between GPs, legally and ethically obligated to provide comprehensive and transparent medical information to the patient, and the patient’s family, who may want to withhold information to protect their relative [16, 17]. This triadic dynamic presents challenges for GPs in Belgium, as they must balance diverse cultural preferences with their legal duty to uphold patient autonomy [15].

Current research on MDM primarily focuses on the dynamics between physicians and patients, overlooking family involvement in the MDM process. Additionally, while studies have mainly concentrated on specialist settings, such as oncology or intensive care [16], relatively little research has examined MDM and family involvement in primary care. However, primary care represents a valuable setting for this research, as GPs are usually the first point of contact within the Belgian healthcare system and provide comprehensive, person-centred care for individuals in the context of their family, community and culture [18]. Despite growing recognition of the importance of family involvement in person-centred care [19–21], empirical research in this field is scarce. It thus remains unclear how personal and cultural preferences and challenges related to MDM manifest among patients with non-European migration backgrounds in Belgium. Identifying these preferences and challenges can inform the adaptation of MDM to better accommodate the needs of patients from diverse backgrounds, ensuring equitable and culturally sensitive healthcare.

This study aimed to broadly explore the diverse preferences and challenges related to MDM among patients with a migration background, from the perspectives of both patients and GPs in Belgium. To achieve this objective, this study employed a qualitative design that includes focus group discussions (FGDs) with patients with a migration background and GPs.

## Methods

### Sample

For both participant groups, we used purposive sampling. Patients were recruited with the aim to achieve maximal variation in gender, professional background and experiences within the Belgian healthcare system. The latter could include both personal experiences as a patient in Belgian healthcare and experiences from other patients in their community, which they may have been witness of through their social and professional activities (e.g., volunteering, informal caregiving, home nursing,…). Hence, both individuals from one of the three major Belgian subpopulations with a migration background (Moroccan, Turkish, and sub-Saharan African) and Belgian organisations that work with patients with a migration background or advocate for culturally sensitive health care were contacted through the researchers’ professional and personal networks and publicly available contact details. GPs were recruited with the aim to achieve diversity in terms of gender, migration background, years of experience, practice location, practice type and payment method. GPs were recruited through the newsletter of the Flemish regional GP association (Domus Medica), and through direct phone or email contact with GPs from the researchers’ network or located via online searches. In addition, a snowball sampling approach was implemented, in which recruited GPs were asked to recommend colleagues for further recruitment.

### Study design

This study employed a qualitative design that involved Braun and Clarke’s reflexive thematic analysis of FGDs [22]. FGDs provided insight into both first- and second-hand experiences of stakeholders and helped identify shared meanings and norms [23]. MDM is typically shaped by interaction between patient and provider [24]. Conducting separate FGDs with GPs and patients allowed us to understand MDM preferences and challenges from both perspectives. The ‘Consolidated criteria for reporting qualitative research’ (COREQ) 32-item checklist [25] was used as a guideline to write this article. The full checklist can be found in Table 1 in the Appendix (pp1-5).

**Table 1:** Participant characteristics of the patient sample (a) and general practitioner sample (b) for focus group discussions held in Antwerp, Belgium and online in November and December 2023

Characteristics	
**a Patient sample**	
Age range (mean)	27–60 (39.4)
Female	19
Country of origin	
Morocco	6
Turkey	7
Sub-Saharan African countries	7^a^
Migration generation	
First generation	4
Years since arrival in Belgium range (mean)	12–57 (34.5)
Second generation	18
Educational level	
Secondary education	3
Higher education	17
Professionally active in the healthcare sector	7
**Total patient participants**	**20**
**b General practitioner sample**	
Female	9
Years of experience range (mean)	2–38 (10.9)
Non-European migration background	1
Urban work setting	10
Group practice	12
Multidisciplinary practice	8
Payment method (fee-for-service)	5
**Total general practitioner participants**	**13**
**Total participants**	**33**

^a^Countries of origin of participants with a sub-Saharan African migration background were Angola, Burundi, D.R. Congo, Gambia, and Mali

### Data collection

Five FGDs, each lasting between 2 and 2,5 h, were held in November and December 2023. Three in-person FGDs with patient participants from Turkish, Moroccan, and sub-Saharan African migration backgrounds respectively were held on campus. The two FGDs with GP participants were held online, via Microsoft Teams. Each FGD was moderated by a junior member and observed by a senior member of the research team. Discussions were held in Dutch and were supported by an interview guide (see Appendix pp5-10). Apart from general questions related to MDM, the interview guide focused specifically on issues related to information disclosure and family involvement, as both the literature and the researchers’ professional experience suggested that these topics were likely to present challenges [26]. Patient participants were asked to discuss experiences (their own experiences as a patient and/or witnessed experiences from others in their community) in which they perceived MDM as challenging, and how patients’ personal and cultural preferences for MDM (both in general and with regards to information disclosure and family involvement) contributed to those experiences. Conversely, GP participants were asked about experiences in which they encountered challenges during MDM with patients with a migration background (both in general and with regards to information disclosure and family involvement). The FGDs were followed by a debriefing between the moderator and observer. Based on audio recording of patient and GP FGDs, ad verbatim transcripts were made. Patient representatives received compensation for their travel expenses and GP participants for their time through consumption vouchers. Conducting five FGDs ensured data sufficiency as the data collected allowed for a broad exploration and comprehensive understanding of a diverse range of perspectives brought in by the participants [27, 28].

### Data analysis

NVivo software (Version 14.23.1) was used to facilitate data analysis. The process of data analysis followed the steps of Braun and Clarke’s reflexive thematic analysis [22]. FV, AY, and NVE each read and re-read all five transcripts to familiarise themselves with the data. Then, for one patient FGD and one GP FGD, FV and AY each created open and inductive codes related to the data describing MDM preferences and challenges. These descriptive codes were then discussed by FV and AY in meetings moderated by NVE and SVDV, with the aim of grouping these codes into preliminary themes. These preliminary themes were four key components of MDM: *exchange of medical information*, *decision-making agency*, *patient-provider relationship*, and *treatment plan*, and guided coding of the remaining transcripts. During the entire coding process, reflexive notes were taken. FV and AY each coded all five individual transcripts and met on multiple occasions to discuss each other’s codes and reflexive notes, with the aim of achieving richer interpretations of meaning [29], encompassing the researchers’ medical and sociological perspectives. Throughout this process, several recurring concepts were identified across the preliminary themes. These concepts represented factors influencing preferences and challenges related to MDM, that explained the variety in observed preferences and challenges depending on the individual and their context. As such, these factors were deemed more coherent as overarching themes. As a result, FV and AY revisited the data and reallocated codes from the preliminary themes to new themes corresponding to these factors. The appropriateness of the new themes was evaluated by comparing their delineation with those of other themes and the codes assigned to each of them. Lastly, final themes were named and defined. To ensure the nuances in the data were captured well, the results were sent to the FGD participants for feedback to take into account during finalisation of the results.

### Ethics

Before the start of each FGD, written consent was obtained from each participant after they had been provided with written information about the study. This research has been approved by the Ethics Committee for Social Sciences and Humanities of the University of Antwerp (file number SHW_2024_142).

## Results

An overview of the study participants’ characteristics can be found in Table 1.

**Fig. 1:**
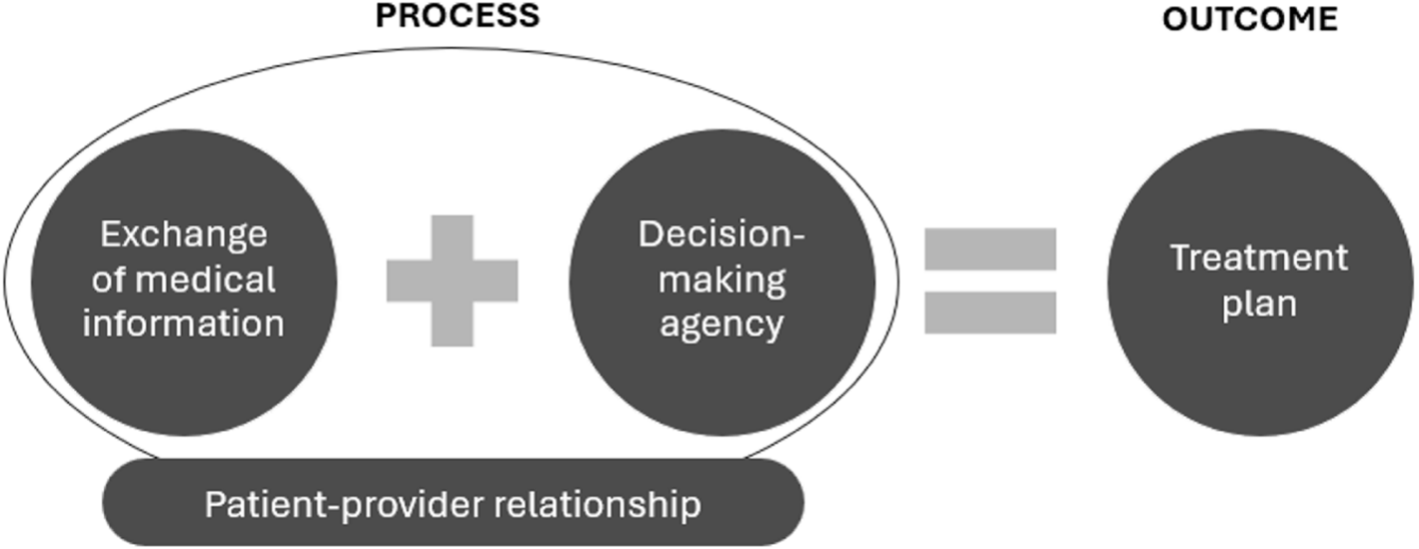
Four key components of medical decision-making and their interrelationship. The four components of medical decision-making, around which medical decision-making preferences and challenges revolved, as identified in the data. Exchange of medical information and decision-making agency were two features of the medical decision-making process, of which the treatment plan was the outcome. The patient-provider relationship was an important contextual factor influencing the medical decision-making process

Asking participants about their experiences with MDM, it became clear that preferences and challenges revolved around four key components of MDM. The first component, *exchange of medical information*, was an umbrella for several actions in the MDM process: on the one hand, healthcare providers communicated details about the diagnosis and treatment options, on the other hand, patients shared their treatment preferences, concerns, values and goals. In addition, when the patient’s family or friends were involved to support the MDM process, it also included the exchange of medical information between patient, provider and family members or friends. Second, *decision-making agency* referred to the role each party (provider, patient and their family and/or friends) played in deciding how to address a health problem, and their ownership over this decision. In order to reach a decision, mutual agreement was sought. Third, the *patient-provider relationship* was characterised by the level of trust and understanding between them. A good relationship allowed patients to express themselves, share concerns and ask questions, and allowed providers to take into account patients’ preferences. It was thus a contextual factor influencing the MDM process. Finally, the *treatment plan* referred to the outcome of MDM. The treatment plan set out how the health issue was going to be dealt with, resulting from decisions on diagnostic testing, preventive measures, treatment and follow-up. Figure 1 illustrates the coherence between the key components.

**Fig. 2:**
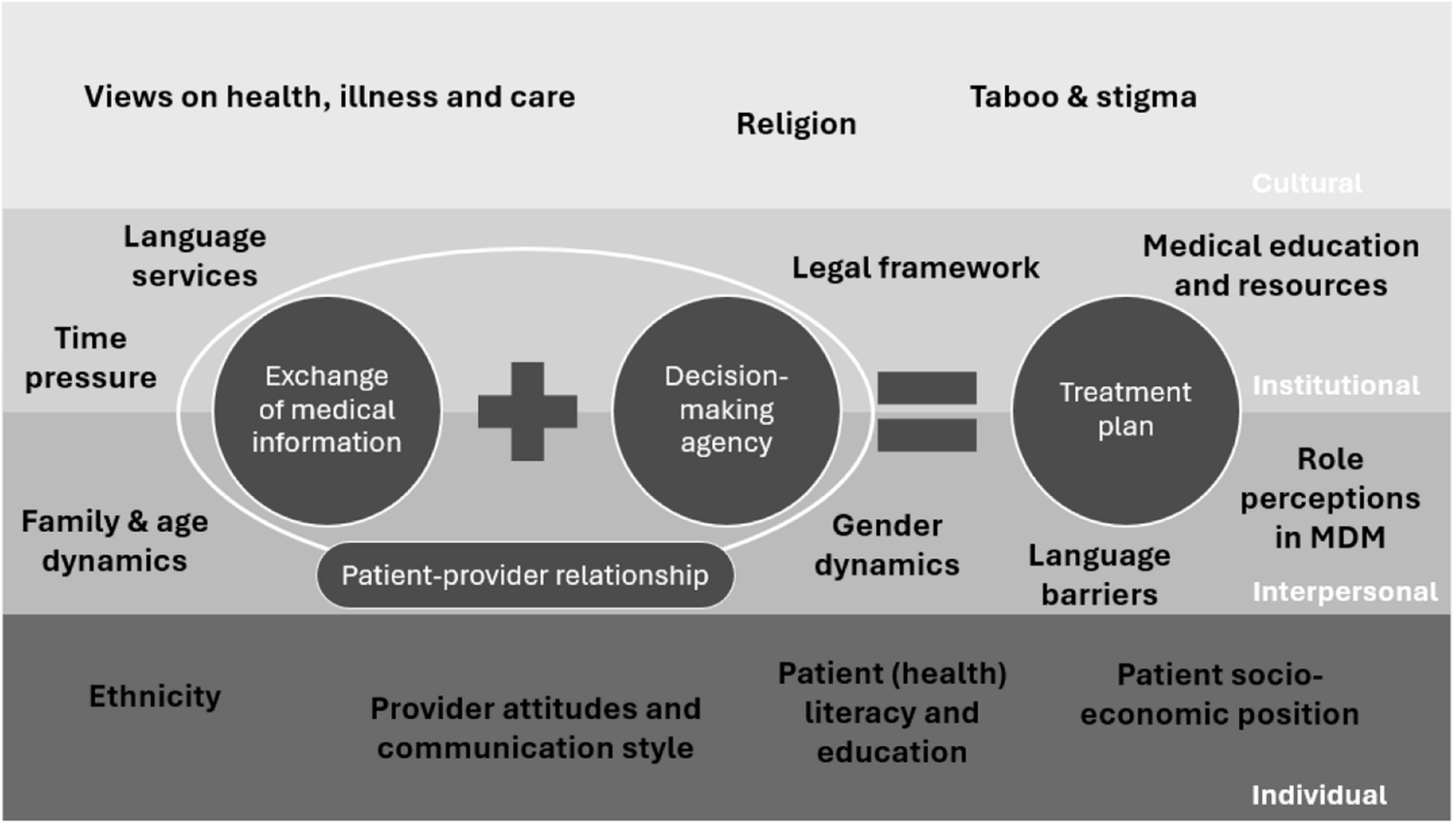
Socio-ecological model of the factors (or themes) shaping preferences and challenges in medical decision-making. The themes identified in the data (in black) are factors shaping the preferences and challenges related to one or several components of medical decision-making, and can be located on different levels of the socio-ecological model (in white, right side of the figure). Abbreviations: MDM: medical decision-making

Participants discussed diverse preferences and challenges related to each of the described four components of MDM. These preferences and challenges were shaped by a complex interplay of factors that could be located at different system levels, including an individual, interpersonal, institutional and cultural level (see Fig. 2). Together, these factors form a socio-ecological model. This model emphasises the role of the social context and the interactions between individuals and their social environment (community norms and values, policies and regulations) in describing and explaining health(care) phenomena [30], including migrant health(care) [31]. These factors were identified in the data as themes, and the mechanisms through which each of these themes affect MDM preferences and challenges, will be explained below. Table 2 summarises which factors (or themes) influence the preferences and challenges for each component of MDM.

**Table 2:** Overview of the factors (or themes) shaping preferences and challenges regarding the medical decision-making components

	Preferences				Challenges			
	Exchange of medical information	Decision-making agency	Patient-provider relationship	Treatment plan	Exchange of medical information	Decision-making agency	Patient-provider relationship	Treatment plan
**Cultural**								
Views	x		x	x	x	x	x	x
Taboo and stigma	x			x	x			x
Religion	x	x	x	x	x	x	x	x
**Institutional**								
Language services	x				x	x		
Time pressure					x	x	x	
Medical education and resources					x	x	x	x
Legal framework	x	x		x	x	x		
**Interpersonal**								
Family and age dynamics	x	x		x	x	x		x
Gender dynamics		x		x		x		x
Language barriers					x	x	x	
Role perceptions in medical decision-making		x				x		
**Individual**								
Ethnicity	x	x	x	x	x		x	x
Provider attitudes and communication style	x	x	x	x				
Patient (health) literacy and education					x	x		
Patient socio-economic status						x		x

The table indicates which factors (or themes) shaped preferences and challenges related to one or several of the four medical decision-making components. A cross indicates that the factor had an impact on specific preferences or challenges with respect to the medical decision-making component

### Cultural factors

#### Views on health, illness and care

Both GP and patient participants discussed that patient and provider may hold different views on health and illness. This complicated the exchange of medical information by hindering mutual understanding when discussing health complaints, and also by affecting the extent to which a patient believed and accepted medical information given by the provider. Their anecdotes showed that patients’ perceptions of some health conditions could differ from the typical biomedical view of the provider. For example, depression and other mental health disorders were associated by some patients with madness or having weak faith:

*A young woman, mentally very ill. […] very religious as well. And she went to a […] hodja [Turkish Islamic religious leader]. And what did they tell her? “Maybe your faith is not strong enough” Instead of telling her: “Miss, you are ill. You should visit a psychiatrist.” (Patient participant 3, Turkish background, FGD 1)*.

Furthermore, dementia and diabetes could be minimised by patients by perceiving them as normal signs of ageing, and there could be different ideas on what body weight was considered healthy. Some diagnoses could also be associated with superstitious beliefs like the evil eye, or could be not believed at all and be considered a ‘white people’s disease’.

Correspondingly, experiences were discussed in which patient and provider held different ideas on what constituted good and appropriate care, shaping preferences and challenges regarding the treatment plan. GP participants’ views were influenced by evidence-based guidelines for clinical practice as well as their own moral principles, with a preference for step-wise treatment to avoid unnecessary care, and prioritisation of preventive measures and lifestyle interventions. On the other hand, patient participants discussed how they and other patients in their community could consider alternative treatment options as complementary or as substitute, e.g., spiritual healing for mental health conditions. Additionally, GP and patient participants’ anecdotes illustrated that some patients were less familiar with chronic disease management, advanced care planning, preventive actions and lifestyle interventions, i.e. measures requiring patients to play an active role in their own health. Rather, these patients preferred that providers handle their health condition through medication and technical examinations. Lastly, patient participants perceived the lack of active treatment of patients suffering from terminal diseases as unacceptable and inhumane.

*I think they give up on patients too soon. […] [For example,] a 37-year-old man with pancreatic cancer, had a bowel perforation. […] They said ‘we can’t do anything anymore’, they sent the man home. No IV, no nothing. ‘Go home, you will die’. […] I think that is inhumane. […] So we had to go for alternative medicine.” (Patient participant 10, Moroccan background, FGD 2)*

Anecdotes from patient and GP participants revealed that patients and providers seemed to have limited insight into each other’s views and struggled to find common ground for decision-making, which compromised the agency of each actor and posed a challenge to their relationship. Participants’ experiences indicated that patients could feel like they were not being taken seriously, which was detrimental to their sense of trust in the provider, increased the chances of requesting second opinions to different providers, and lowered treatment adherence by the patient. Because of that, some patient participants expressed a preference for providers who shared their cultural background.

*I understand the mistrust, care is organised differently here… What [patients] see as good care, we might not be offering. We prescribe no antibiotics, too little for them. We sometimes prescribe too little scans […]. Maybe for them it’s the way of […] being recognized that those problems are actually there. (GP Participant 11, Belgian background, FGD 5)*

#### Taboo and stigma

Patient and GP participants discussed the presence of taboo and stigma within ethnic minority communities, although patient participants mentioned that its impact was lower among younger generations, having a changing mindset and upbringing, as well as higher education. Provided examples primarily pertained to mental health and sexual and reproductive health.

GP and patient participants’ anecdotes showed that taboo and stigma complicated receiving and sharing information about conditions affecting mental health and sexual and reproductive health. Patients could feel hesitant or unable to discuss these matters with their provider, particularly when the provider had the same cultural background. Furthermore, they could question or repudiate information from the provider. Patient participants also preferred not to share such health issues with their family or friends. Interestingly, patient participants with a sub-Saharan African background also noticed that Belgian providers could be hesitant to discuss female genital mutilation.

Moreover, patient and GP participants’ anecdotes illustrated that taboo and stigma made it challenging to agree on a treatment plan. Treatments advised by the provider could be considered unacceptable by the patient: taboo limited knowledge about appropriate treatment and reduced help-seeking behaviour, and by avoiding treatment, patients tried to avoid the stigma linked to these conditions.

*The first generation [migrants], when they have an issue with something and we ask them “Shall we consult a psychologist together?” They don’t want to hear anything about it, because seeing a psychologist immediately means that you are crazy. You would get a label. (Patient participant 6, Turkish background, FGD 1)*

#### Religion

Experiences discussed by both patient and GP participants illustrated how religion could affect the exchange of information, their decision-making agency and the treatment plan. Religious influence was most evident in decisions pertaining to the beginning of life (reproductive healthcare) and end-of-life care. Both stages of life were recognised as a process that was ultimately in God’s hands, and it was deemed inappropriate for the patient or the provider to intervene in this process. Therefore, abortion (and, for some patient participants, prenatal diagnosis of genetic diseases by extension), euthanasia and discontinuation of life support (or any treatment that was perceived to extend life) were considered undesirable for patient participants who were religious. Some of them, in their professional role as nurses, also preferred to avoid providing such treatment options to their patients. However, varying interpretations of religion lead to variations in these preferences. Anecdotes from GP and patient participants illustrated that the preference to exhaust all treatment options and extend life as much as possible seemed less prevalent among some patients with a strong belief in fate. Such patients could be convinced that the outcome of their situation had already been predetermined by God and therefore believed that prevention and treatment, such as early cancer detection and treatment, could not make a difference. On the other hand, fate helped patients cope with bad news. As such, fate was seen by the GP participants as both a facilitator and a barrier for the MDM process.

*[I had] a call this week from a patient who had had a positive screening test for colorectal cancer, and the patient refused [further diagnostic testing]. I asked him why, and he said “It is my fate and I have to accept it”. (GP Participant 6, Belgian background, FGD 4)*

*[The participant’s mother said] “God gave me this cancer, so I shall live with it. I don’t want to get surgery.” […] She didn’t believe in [cancer] treatment either. You would say [to her] “you have cancer” but she would simply laugh it away. – She would say “well, those doctors aren’t God”. (Patient participants 4 and 5, Turkish background, FGD 1)*

A related topic was pain relief in the final stages of life. Participants explained that some religious patients viewed suffering as a way to achieve remission of sins, rather than something to be avoided at all costs. In addition, some religious patients found it crucial to appear conscious to God when transitioning into the afterlife. Hence, as some pain medications can sedate patients at certain doses, they were generally avoided by these patients. Furthermore, morphine could be perceived by patients as a means of hastening death, especially among those with low health literacy (see also “Patient (health) literacy and education” section). These considerations were also mentioned as reasons why some patients or family members refuse palliative sedation.

*We make sure the patient’s pain is bearable but you can never take the pain away completely without sedation. But they cannot sleep because they need to be conscious to […] beg forgiveness or make supplications to Allah. (Patient participant 10, Moroccan background, FGD 2)*

In general, patient participants wanted the provider and the treatment plan to respect their religious principles, but often encountered challenges in having this preference met:

*At a certain point [the gynaecologist] said “I don’t understand why someone as articulate and intelligent as you would settle for a child with disability. And then I really flipped out and said “Look, if I were allowed to choose, I wouldn’t. But I have no choice, and I accept what I am given.” And [the gynaecologist said] “Sure, and why would you stay pregnant if there is a [high risk] that your child will decease?” And then I said “Doctor, neither you nor I can decide about death and life. I have someone else for that.” (Patient participant 12, Moroccan background, FGD 2)*

This worsened their relationship with the provider, causing some patient participants to prefer sharing a religious background with their provider. Another challenge indicated by patient participants referred to providers mistakenly refraining from suggesting treatment they deemed unacceptable to a patient for religious reasons.

### Institutional factors

#### Language services

GP participants preferred professional language services (interpreters or intercultural mediators) to ensure accurate translation, especially for complex or sensitive information. In addition, this allowed family members and friends to resume their customary roles, rather than having to voice both the patient’s and their own perspectives. Nevertheless, in some cases discussed by GP participants, professional language support was rejected by family members of the patient. Moreover, some GP participants reported inappropriate behaviour of some professional interpreters as they took over the consultation. Above all, they mentioned that there was generally a lack of time and resources to guarantee professional language services for each consultation where a language barrier appeared, especially for home visits.

*We preferred to explain the new diagnosis [hypothyroidism] and consequences in [the patient’s] own language. […] It went wrong because the intercultural mediator had a double booking. So we planned a new consultation with an intercultural mediator. The next week, the intercultural mediator was there, but [the patient] wasn’t. (GP participant 9, Belgian background, FGD 5)*

Therefore, GP participants often preferred lay interpreting by family members or friends due to its convenience, especially when compared to translation apps or websites. However, GP and patient participants noted that lay interpreters, whether knowingly or unknowingly, could filter communication between patients and providers, causing information and emotions to be conveyed incompletely or incorrectly in both directions:

*I ask the question “do you have to vomit?”. Then the [lay] interpreter asks the question, which lasts five minutes. [The patient’s] explanation takes ten minutes. And then the answer is “yes”. Then I’m thinking like “what have you actually been saying to each other the past ten minutes?” (GP participant 10, Belgian background, FGD 5)*

*We [children of migrants] had to act as translators as a child at the gynaecologist [when our parents had to visit them]. I mean, are you crazy? ‘Does it hurt during intercourse?’ What?! How was I supposed to ask that to a woman [the patient]. I didn’t even know what that was. (Patient participant 3, Turkish background, FGD 1)*

This could diminish the decision-making agency of the patient. Participants also questioned the extent to which lay interpreters were allowed to hear sensitive information being shared in the consultation room, although GP participants’ anecdotes showed that some patients felt uncomfortable discussing taboo or stigmatised matters even with a professional interpreter, due to the common cultural or religious background.

#### Time pressure

Participants acknowledged that taking the time to communicate properly helped to build a trusting relationship, increase mutual understanding and find common ground for decisions on treatment. However, anecdotes from all FGDs showed that healthcare providers frequently faced significant workloads and time constraints.

#### Medical education and other resources for culturally sensitive healthcare

In order to anticipate diverse patient preferences and provide quality care, provider medical education and access to other resources to support culturally sensitive healthcare was seen as critical, but currently inadequate, both by GP and patient participants. Although GP participants were aware of the diversity among their own and their patients’ backgrounds, they indicated not always being able to understand and manage differences, which challenged the MDM process and affected trust. In cases where the treatment plan required tailoring to religious or cultural practices, e.g. diabetes management during Ramadan, additional guidance was needed.

*In terms of [prescribing] medication, I didn’t know what to do. But actually, I also found I simply didn’t know enough about Ramadan itself to give advice about it. (GP participant 12, Belgian background, FGD 5)*

Patient participants indicated that providers sometimes insufficiently accommodated their preferences and needs, for which they blamed providers’ education and the prevailing policies and guidelines in Belgian healthcare, such as requirements to undress for examinations.

*Physicians are insufficiently informed about our values. If they know we believe in fate, I think there will be more understanding. (Patient participant 10, Moroccan background, FGD 2)*

#### Legal framework

Both patient and GP participants seemed to be aware of patient rights and their relevance to the MDM process. For the patient participants, patient rights empowered them in the pursuit to have their information and treatment preferences met.

*You always have the right to a second opinion. […] you don’t need to stay with one doctor. […] always need to follow your own choice. (Patient participant 9, Moroccan background, FGD 2)*

For the GP participants, patient rights were guiding principles for the exchange of medical information and balancing decision-making agencies. However, in some discussed cases, discrepancies between the legal framework and patients or families’ preferences for MDM were evident and challenging to resolve.

*[The patient with cancer] has the right to know of course. But yeah, he does not understand Dutch. Only what [his] children translate, and they say “we won’t go see a specialist, because he does not want all of that.” But actually, I don’t know what the man wants. Because he cannot say what he wants. And I found it really difficult that he didn’t know he had cancer. (GP participant 10, Belgian background, FGD 5)*

### Interpersonal factors

#### Family and age dynamics

Participants discussed how family members often wanted to care for one another, especially for older persons, and protect each other from worries and distress about their health. They described cases where such mechanisms of reciprocal protection and care influenced exchange of information and agency during MDM, as well as the treatment plan.

Anecdotes showed how family members may participate in the exchange of information between patient and provider, affecting this process in several ways. With regards to patients and family sharing information with the provider, GP participants appreciated how information given by family members increased their insight into the patients’ situation. However, they also acknowledged a risk that family members were divulging information that patients preferred to withhold from the provider, and a risk that patients were not comfortable discussing certain issues in presence of their family. With regards to providers sharing information with the patient and family, providers could also be sharing information that the patient did not want their family to know. Patient participants indicated that their family members’ emotional responses could cause them additional distress. Vice versa, both GP and patient participants’ anecdotes illustrated that sometimes family members did not want the provider to share a stressful diagnosis with the patient, as they believed that learning about such information could harm the patient’s wellbeing. They could prefer the patient to be unaware about their diagnosis (or only partially, e.g. not use the word ‘cancer’), or prefer disclosing this information themselves in a calmer and more trusted environment.

*My mother […] she was terminal […], the doctor wanted to tell my mother […] We couldn’t tell her. You know your mother better than the doctor does. You know what she can handle. (Patient participant 3, Turkish background, FGD 1)*

However, providers were often not comfortable engaging in such non-disclosure practices and preferred honest and open communication.

*A woman with cancer. The children were translating and the woman was absolutely not allowed to know she had cancer. And that was very difficult to deal with. They refused making use of an intercultural mediator. It is impossible to estimate why they do not want to give the information. (GP participant 13, Belgian background, FGD 5)*

Furthermore, anecdotes showed that family members may have decision-making agency by offering advice to the patient (particularly if they are healthcare professionals themselves), expressing wishes or opinions, or advocating (or even deciding) on the patient’s behalf. Following the consultation, they could provide support and help patients adhere to treatment, which was a reason for GP participants favouring family involvement. However, GP participants were hesitant about the extent to which family involvement was conducive to a patient-centred MDM process, as family members’ intentions were often difficult to interpret:

*A girl […] got pregnant at 15. Her first visit was already with her mother-in-law, […] and abortion was absolutely out of the question. […] So that is very difficult. Everyone is involved, happy with the baby… but where is the shared decision-making in that moment? (GP participant 11, Belgian background, FGD 5)*

Similarly, patient participants demonstrated ambivalence towards family involvement: although appreciating family support, they also indicated that the advice they received was occasionally unsolicited and challenging to disregard.

Finally, (elderly) care in the community, as opposed to institutionalised care, was considered the norm among patient participants. However, they discussed that this norm proved challenging to uphold in practice, as women, often the primary caregivers, were increasingly participating in the labour market.

#### Gender dynamics

Preferences for gender concordance between patient and provider were expressed among patient participants from all backgrounds and recognised by GP participants, especially for, but not limited to intimate examinations. Anecdotes from GP and patient participants illustrated that this preference could not always be met, which led patients to postpone their care, and that, in some cases, husbands prevented their wives from receiving care from a male provider.

*We had two male and two female GPs [in our practice]. But whenever the other female GP was fully booked, and I [a female GP] was on holiday, the female patients were absolutely not allowed to see the male GPs in our practice. That was a very big problem. Even when there were urgent issues, it wasn’t an option at all. Often, it wasn’t allowed by the husband or partner. (GP participant 12, Belgian background, FGD 5)*

With regards to decision-making agency, patient participants with a Turkish background discussed that their male family members, who were perceived as more rational, needed to be involved in major medical decisions, such as those about surgery or end-of-life care. Their anecdotes also illustrated that because of a patriarchal family dynamic, a female patient’s husband and mother-in-law may have significant influence on medical decisions concerning her health (e.g. by attending the patient’s visit to the gynaecologist).

#### Language barriers

Anecdotes from both GP and patient participants illustrated that the lack of a common language between patient and provider posed a tremendous challenge for all three components of the MDM process. Patients could not express their questions, concerns, wishes nor treatment preferences. This led to less mutual discussion and less patient participation in the MDM process. As a result, family members, the provider, or both, could have increased authority over the final decision.

*There are very often situations with a language barrier. […] Then often a lot of decisions are made on behalf of [the patients]. For instance, pregnant patients who come for a prenatal genetic screening test. […] Then [physicians] say “you should do it”, whereas, no, you don’t have to do that. Why should [someone] know beforehand whether [the baby] will have a severe handicap? [Physicians] may urge [them] to do something that does not align with their religious principles or cultural values. (Patient participant 18, Sub-Saharan African background, FGD 3)*

With a language barrier, some GP participants also experienced challenges in conveying empathy and thus, found it more difficult to build trust.

#### Role perceptions in MDM

The experiences discussed by the participants indicated that the eventual roles that patient and provider had in the MDM process, i.e., their decision-making agency, also depended on their perceptions of these roles. These role perceptions varied depending on the nature of the decision: e.g., GP participants preferred patient participation in MDM more for chronic than for acute diseases. In practice, patient roles seemed to vary from obedient to demanding, as illustrated by examples from all FGDs. GP participants attributed their patients’ agency preferences (at least in part) to the standard (as perceived by the GPs) in their patients’ home countries. GP participants, although exhibiting some variation within the sample, indicated a preference for engaging in a mutual discussion about decisions, while still maintaining ownership over the final decision. However, they did not always manage to muster the energy to counter demands from patients or their families, even when they disagreed with them. Anecdotes from GP and patient participants revealed that, when MDM roles were out of balance, it could result in dissatisfaction with the quality of care on both sides. This could lead to frustration among providers and reduced adherence among patients.

*That was a patient [with overweight and poorly controlled diabetes] who had done a lot of technical examinations, e.g. scans for her back, because she absolutely wanted that. I always tried to hold that off a bit, but then after a while it didn’t work. [...] In this way, a lot of time and energy was actually lost in that kind of discussion. So they were not about the essence of the matter - in my opinion. (GP participant 3, Belgian background, FGD 4)*

### Individual factors

#### Ethnicity

Particularly participants with a sub-Saharan African background mentioned that the treatment plan (diagnostics and therapy) should be tailored to their ethnicity, but that, in their perception, the standard of care in Belgium did not always take this into account. The (perceived) lack of knowledge of Belgian providers about these ethnicity-specific considerations in healthcare had an impact on the MDM process: patient participants’ anecdotes revealed that diagnostic information and therapeutic advice from native Belgian providers was received by patients with caution, and often double-checked by providers from their own community. Some patient participants felt the need to educate themselves, and wanted to have a larger say in MDM, increasing their agency to ensure their care was tailored to their ethnicity.

*[Doctors say] “yes but I do think it will be better for you if you...” […] yes you may know, and this may be very crudely put, you know [what’s best] for the white population but for me you may not know. We are not necessarily the same as the white population. (Patient participant 14, Sub-Saharan African background, FGD 3)*

As discussed in the "Views on health, illness and care" and "Religion" sections, patient participants from all backgrounds acknowledged the benefit of ethnic concordant care, i.e. a shared cultural and/or religious background between patient and provider. However, the following quote illustrates that this preference varied depending on the situation, as mentioned in the "Taboo and stigma" section.

*I think we can all agree that mortality in births is much more complicated for Black people than for white people. So that we as Blacks are really very often these days sitting around asking: “Is anyone a Black gynaecologist? A Black midwife? A Black this a Black that, an African this?” So it’s the trust, but also the distrust if something goes wrong. [...] Then you want that white person. Then you would rather have a white person who doesn’t know me, who is going to keep their mouth shut. (Patient participant 14, Sub-Saharan African background, FGD 3)*

#### Provider attitudes and communication style

Participants in all FGDs also discussed the importance of the provider’s communication style and attitudes towards the patient, beyond the importance of ethnic concordant care. They preferred an empathetic, human, and person-centred approach towards the patient, in which providers communicated respectfully and showed consideration for the patient’s perspective, even when it differed from their own. According to patient participants, this included making efforts to overcome the language barrier and to adapt care in order to meet the needs of a patient. Such behaviours improved the patient-provider relationship by building trust, which in turn made it easier to exchange medical information and to come to a mutual agreement when deciding upon a treatment plan:

*My father is a diabetic. [He] has basically always been told, “May not fast during Ramadan. May not. May not. May not.” However, [he] has always done so, because […] the value of fasting is more important to him, than his pills. [...] Whereas those GPs never looked for alternatives. [...] When we got new GPs, [...] that GP knew that [my father] was going to [fast] anyway, so he was looking for... “Okay, in what way can I support this?” [...] From the moment he got that advice “Okay, you can fast but I’m going to guide you”, [my father] also started accepting other [advice] much more easily from that doctor. And [he would] not like [say anymore], [...] “he doesn’t understand us anyway.” (Patient participant 11, Moroccan background, FGD 2)*

#### Patient (health) literacy and education

Health literacy of the patient, as well as literacy and educational level, were discussed by GP and patient participants as crucial aspects affecting MDM. Their anecdotes revealed how exchanging medical information became challenging when patients had a lower degree of understanding of their condition and appropriate treatment options, which in turn reduced their agency in decision-making, and lowered treatment adherence. Patient participants, all relatively highly educated, discussed how they wanted to be involved in medical decisions and would stand up for themselves, but acknowledged that many individuals with the same migration background were not in the same position. Challenges of low (health) literacy and education seemed to decrease in younger generations.

*We have studied here, we know what is going on. But our parents, who have studied less, and low-educated people. – Patient participant 5: My mother [did] not [study] at all. She couldn’t read actually. – So we do know what a psychologist is and what a psychiatrist can do for us. But our parents just don’t know. (Patient participant 2, Turkish background, FGD 1)*

#### Patient socio-economic position

Although often linked to challenges of low health literacy, participants discussed how patients’ socio-economic position also posed challenges in terms of decision-making agency and the treatment plan. On the one hand, GP participants experienced that patients with a low socio-economic position had relatively limited agency, considering their health as something they could not have an impact on themselves. In the GP participants’ experience, this led some patients to prefer treatment options of which effectiveness was independent of their own efforts, and to prioritise acute complaints, giving less attention to prevention and chronic disease management.

*We do need to distinguish between the causes as to why people attach little importance to prevention? And we then look immediately at cultural differences, but I don’t think that only a cultural difference explains that. I think much more important, for instance, is the person’s socio-economic status and health literacy. And I notice that if people score low in that, that regardless of the culture to which they belong, they actually attach less importance to prevention. (GP participant 4, Moroccan background, FGD 4)*

On the other hand, agreeing on a treatment plan could also be challenging because of the financial accessibility of treatment options.

## Discussion

The aim of this study was to explore the preferences and challenges related to MDM among patients with a migration background from the perspectives of patients and GPs in Belgium. To the best of our knowledge, this study is the first qualitative research in Belgium to address this topic from both patients’ and GPs’ perspectives. We can identify three main conclusions based on these results.

First, our study showed that preferences and challenges in MDM relate to four key components of MDM: exchange of medical information, decision-making agency, patient-provider relationship, and treatment plan. The exchange of medical information is crucial in the MDM process. Existing MDM models underscore the importance of mutual information sharing, during which patients express their preferences, to ensure the patient’s voice is heard [13]. They focus on the patient-provider dyad, as highlighted in studies by Djulbegovic et al. [32] and Légaré and Witteman [33]. Despite significant evidence on the importance of family involvement in managing serious illnesses [34], many models still overlook family members as key participants in MDM. Our research indicates that such traditional MDM models are insufficient, especially in diverse societies where decisions often involve family, as noted by de Pentheny O’Kelly, Urch, and Brown [17]. Moreover, community leaders, religious figures, and social networks can add various perspectives, values, and expectations to the MDM process. The relationship between the patient and the healthcare provider is crucial in most studies about MDM. It helps build trust and allows patients to communicate their preferences [13]. The treatment plan may be the most vital part of MDM. It is often viewed as a result of mutual agreement between patient and provider, discussing medical information, healthcare outcomes, and treatment options [35].

Second, our findings demonstrate that it is impossible to reduce the MDM process to the patient’s individual context. Factors such as views on health, illness and care, religion, taboo and stigma, legal framework, language services, time pressure, medical education and other resources for culturally sensitive healthcare all shape the MDM process. These factors can be effectively understood using the socioecological model, as supported by previous literature, where the key influences on MDM exist across several levels: individual, interpersonal, institutional, and societal [36, 37]. Our results indicate that the decision to involve family in MDM varies based on several factors. These include family dynamics, the severity of the illness, potential overreactions by family members, and the presence of any taboos or stigmas. This is particularly relevant in culturally diverse populations, where preferences for self-determination and family involvement vary widely [36]. In some cultures, family input is prioritised, while in others, patient autonomy takes precedence [38]. Additionally, legal frameworks and healthcare policies contribute to the institutional complexity of the MDM process [15], and the implementation of MDM models varies across countries [39]. Furthermore, research shows that healthcare policies and legal frameworks regarding integration contribute to healthcare inequalities between patients with a migration background and those without [40, 41].

Finally, our results highlight the significance of taking an intersectional approach to MDM. A patient’s age, gender, ethnicity, health literacy, education, socioeconomic position, role perceptions in MDM, language proficiency, and family dynamics all influence their preferences and challenges in MDM. GP participants also stressed the importance of not treating the patient population with a migration background as a homogeneous group. These findings align with previous studies showing that individual factors influence preferences for family involvement in healthcare [38, 42]. These factors, both personal and contextual, affect health, illness, and the approach to care [41]. As such, research has highlighted the importance of prioritising person-centred care over culturally sensitive care to avoid the risk of stereotyping [38]. Moreover, studies suggest that patients with a migration background in Europe face challenges when communicating with healthcare providers due to factors such as age, language barriers, education, literacy, and perspectives on MDM [43, 44]. Additionally, family dynamics, gender, generation, and migration status significantly influence MDM among patients with a migration background in Belgium [39, 45]. Finally, research indicates that socioeconomic inequalities impact the utilisation of GP services among financially vulnerable individuals despite increased reimbursement policies [41].

### Implications

Our study highlights the importance of adopting an intersectional and multilayered perspective on MDM, which aligns with the WHO European Review on Social Determinants of Health. The WHO emphasises that various social, economic, and environmental factors influence health outcomes [46]. This highlights the need to address systemic health behaviours and care access inequalities, ultimately shaping MDM processes. Moreover, while it may not be possible to be aware of every cultural preference, GPs can adopt a more culturally sensitive approach in their interactions with patients. To this end, it is essential for GPs to receive comprehensive training in person-centred communication, with additional emphasis on the underlying factors that shape patient-provider interactions. This effort creates greater understanding and respect for patients’ individual and cultural preferences during MDM, promoting more equitable and person-centred care, while also taking into account the needs of the provider.

### Study limitations

Our study concentrated on the three major population subgroups with a migration background in Belgium. However, we did not compare our findings with the preferences and challenges in MDM among the general population. Therefore, we cannot claim these results are unique to people with a migration background. Moreover, it is important to note that GPs referred to patients with migration backgrounds including but not limited to Moroccan, Turkish, or sub-Saharan African, because their experiences showed relevant challenges. Furthermore, we acknowledge that we may not have been able to fully capture the perspectives of individuals born outside of Belgium who later migrated. Future research could enhance this work by including the voices of first-generation migrants for a more comprehensive view. Additionally, although we aimed for greater diversity in our sample of GPs, our purposive sampling approach did not fully realise this goal. We recruited more GPs without a migration background and encountered a disproportionately high number of GPs working under a capitation payment system. Additionally, our sample included more GPs from group practices and fewer from individual practices. However, we were able to include GPs with significant experience working with patients from migration backgrounds, making their insights particularly valuable. Finally, the study sample of patient representatives was skewed towards individuals with higher levels of education and a greater proportion of women, which may have introduced bias into our results.

## Conclusions

Preferences and challenges regarding MDM among patients with a migration background in Belgium are shaped by a myriad of factors, existing across various levels of the socio-ecological model. The findings of this study enhance our understanding of the complexities surrounding MDM in this setting. Insights can assist healthcare providers in accommodating the unique needs of each patient during MDM and guide policymakers in addressing institutional factors that contribute to challenges in MDM. Such comprehensive measures are vital to promote high-quality, person-centred care for all.

## Supplementary Information

Supplementary Material 1

## Data Availability

The data generated and analysed during the current study, in the form of pseudonymised transcripts in the Dutch language, can be discussed upon reasonable request with the corresponding author.

## Abbreviations

FGD: Focus group discussion
GP: General practitioner
MDM: Medical decision-making
WHO: World Health Organization
