# Supplementary material for: Preferences and challenges regarding medical decision-making among patients with a migration background in Belgium: a focus group study
Source: Arch Public Health. 2025 Jul 1;83:167. doi: 10.1186/s13690-025-01648-7 (PMC12210460; doi:10.1186/s13690-025-01648-7)
Supplement: Supplementary file 1 — Supplementary Material 1 [file 13690_2025_1648_MOESM1_ESM.docx]

# Appendix

**Table 1.** Application of the “Consolidated criteria for reporting qualitative studies (COREQ): 32-item checklist” to this study.

| No | Item | Guide questions/description | Explanation |
| --- | --- | --- | --- |
| **Domain 1: Research team and reflexivity** | | | |
| Personal Characteristics | | | |
| 1. | Interviewer/facilitator | Which author/s conducted the interview or focus group? | GP FGD1: FV (supported by NVE and JVO)  GP FGD2: FV (supported by KB)  Patient representative FGD (Moroccan): AY (supported by EW and FV)  Patient representative FGD (sub-Saharan African): AY (supported by SVDV)  Patient representative FGD (Turkish): AY (supported by NVE) |
| 2. | Credentials | What were the researcher's credentials? E.g. PhD, MD | AY, Master in Sociology  FV, MD, MSc  KB, MD, PhD  VB, PhD  SVDV, PhD  JVO, MD, PhD  PVR, MD, PhD  EW, PhD  NVE, PhD |
| 3. | Occupation | What was their occupation at the time of the study? | AY and FV: full-time PhD researcher  KB, JVO, and PVR: part-time general practitioner and part-time professor  VB, SVDV, EW: full-time professor  NVE: postdoctoral researcher – coordinator of the MEDIMIG project |
| 4. | Gender | Was the researcher male or female? | AY, FV, KB, JVO, VB, SVDV, NVE: female  PVR, EW: male |
| 5. | Experience and training | What experience or training did the researcher have? | FV and AY were junior researchers, trained during their respective research master’s programmes. FV has additionally been trained in person-centred communication. |
| Relationship with participants | | | |
| 6. | Relationship established | Was a relationship established prior to study commencement? | GP FGDs: no relationship established  Patient representative FGDs: superficial professional relationship established with a minority of the participants. |
| 7. | Participant knowledge of the interviewer | What did the participants know about the researcher? e.g. personal goals, reasons for doing the research | The participants were informed about the research aims and the interviewer’s position through the informed consent letter. At the start of the FGD, the interviewer introduced themselves to all participants. |
| 8. | Interviewer characteristics | What characteristics were reported about the interviewer/facilitator? e.g. Bias, assumptions, reasons and interests in the research topic | FV is a medical doctor by training and has brought a medical perspective to the analysis. As a GP trainee, she has received training in person-centred communication, and has a personal interest in working with a culturally diverse patient population. She was born in Luxemburg, grew up in Belgium, and has a Belgian-Dutch background. AY is a medical sociologist by training and has brought a sociological perspective to the analysis. She was born in Belgium, grew up there, and has a Moroccan background. AY is interested in equality in healthcare and patient-centered care. |
| **Domain 2: study design** | | | |
| Theoretical framework | | | |
| 9. | Methodological orientation and Theory | What methodological orientation was stated to underpin the study? e.g. grounded theory, discourse analysis, ethnography, phenomenology, content analysis | Reflexive thematic analysis |
| Participant selection | | | |
| 10. | Sampling | How were participants selected? e.g. purposive, convenience, consecutive, snowball | Purposive sampling |
| 11. | Method of approach | How were participants approached? e.g. face-to-face, telephone, mail, email | Patient representative FGDs: face-to-face, telephone, email, LinkedIn  GP FGDs: telephone, LinkedIn, email |
| 12. | Sample size | How many participants were in the study? | 33 participants |
| 13. | Non-participation | How many people refused to participate or dropped out? Reasons? | For GP FGDs: a total of 44 GP practices, with a total of 160 GPs, were contacted, of which 14 agreed to participate. Refusal to participate was because of time constraints. One GP dropped out last-minute because of similar reasons.  For patient representative FGDs: a total of 46 individuals and 34 organisations were contacted. 30 persons agreed to participate. Refusal to participate was because of time, practical reasons, such as too far, etc. One person said she didn’t feel comfortable to share her personal expierences of opinions in a group. 10 participants dropped out last minute because of being sick, illness of a child, an emergency at work, etc. |
| Setting | | | |
| 14. | Setting of data collection | Where was the data collected? e.g. home, clinic, workplace | For the patient representative FGDs, data was collected in meeting rooms of the University’s campus. The GP FGDs were organized online, where participants could participate from their preferred setting. |
| 15. | Presence of non-participants | Was anyone else present besides the participants and researchers? | No. |
| 16. | Description of sample | What are the important characteristics of the sample? e.g. demographic data, date | GP participants (n=13) were mostly female (n=9), working in a group practice (n=12) in an urban setting (n=10), and paid under capitation (n=8). Their experience as a GP ranged from 2 to 38 years (mean of 11 years), and one GP had a migration background (Moroccan). Patient representative participants (having a Moroccan (n=6), Turkish (n=7) and sub-Saharan African^[[1]](#footnote-2)^ (n=7) migration background), were mostly female (n=19), with a second-generation migration background (n=16). Their ages ranged between 27 and 60. The majority received higher education (n=17), and a third worked in the healthcare sector (n=7). An overview of the study participants’ characteristics can be found in Table 2 in the Appendix. |
| Data collection | | |  |
| 17. | Interview guide | Were questions, prompts, guides provided by the authors? Was it pilot tested? | See below in the Appendix. The guides have been pilot tested within the research group. Furthermore, after the first FGD with each participant group, some small refinements were made to the interview guide. |
| 18. | Repeat interviews | Were repeat interviews carried out? If yes, how many? | No. |
| 19. | Audio/visual recording | Did the research use audio or visual recording to collect the data? | The patient representative FGDs were audio-recorded, the GP FGDs were video-recorded in Microsoft Teams. |
| 20. | Field notes | Were field notes made during and/or after the interview or focus group? | Yes, by the co-moderator and during debriefing between the moderator and co-moderator. |
| 21. | Duration | What was the duration of the interviews or focus group? | GP FGDs lasted 2 hours.  Patient representative FGDs lasted between 2 and 2,5 hours. |
| 22. | Data saturation | Was data saturation discussed? | Yes, see main text. Conducting five FGDs ensured data sufficiency as the data collected allowed for a broad exploration and comprehensive understanding of the participants’ perspectives. |
| 23. | Transcripts returned | Were transcripts returned to participants for comment and/or correction? | The transcripts were not returned, the results of the data analysis were sent to the participants for their comments and feedback (see 28). |
| **Domain 3: analysis and findings** | | | |
| Data analysis | | | |
| 24. | Number of data coders | How many data coders coded the data? | 2 (AY and FV) |
| 25. | Description of the coding tree | Did authors provide a description of the coding tree? | Yes, see under ‘2.4 Data analysis’ in the Methods section of the main text. |
| 26. | Derivation of themes | Were themes identified in advance or derived from the data? | The themes were derived from the data, applying an inductive coding process. |
| 27. | Software | What software, if applicable, was used to manage the data? | NVivo Version 14.23.1. |
| 28. | Participant checking | Did participants provide feedback on the findings? | All participants were sent an email and given three weeks to provide their feedback on the findings (both the original version and a Dutch translation of it). Participants responded with mostly positive feedback, agreeing with the findings. Some of them had substantial feedback, which was taken into account in the final manuscript. |
| Reporting | | | |
| 29. | Quotations presented | Were participant quotations presented to illustrate the themes / findings? Was each quotation identified? e.g. participant number | Yes. |
| 30. | Data and findings consistent | Was there consistency between the data presented and the findings? | This was intended by the authors and thoroughly discussed within the team. |
| 31. | Clarity of major themes | Were major themes clearly presented in the findings? | This was intended by the authors and thoroughly discussed within the team. The socio-ecological model was found most applicable to clearly present the themes. |
| 32. | Clarity of minor themes | Is there a description of diverse cases or discussion of minor themes? | Not applicable. |

Abbreviations: FGD: focus group discussion, GP: general practitioner.

### Focus group discussion with general practitioners: interview guide

**Homework for the participant (sent in advance via email):**

Can you tell about an experience with a patient from a non-Western migration background, where you had to make a decision about his/her care during the consultation and this was a difficult process? This decision may have been made together with the patient and/or his/her family, but this is not a requirement.

**Introduction:**

- Hello, welcome all to this focus group. Before we get started, I will explain a bit about the project and some practical things about the course.
- We are (names, background (sociologist, GP-in-training, GP-researcher...)), working together on an interdisciplinary research project on medical decision-making in patients with non-Western migration background in the European context.
- The ultimate goal of this project is to develop recommendations and tools that can support healthcare providers in diversity-sensitive medical decision-making. Medical decision-making is about the stage in the consultation where you come to a plan, for example about further investigation or treatment, or a plan not to do such things. So they are decisions about care that you make during the consultation, with or without your patient. So what do we mean by culturally-sensitive or diversity-sensitive medical decision-making? That is about the extent to which, as a healthcare provider, you can adapt the decision-making process to the patient's preferences around decision-making, which may or may not be culturally influenced. With the tools developed, we hope that the work of healthcare providers can be facilitated. So we are happy to listen to the needs of GPs like you tonight, but also in the years to come.
- This focus group is part of the first phase of this project. In it, we want to get a picture of all possible cases or situations that are challenging for GPs in terms of the medical decision-making process in patients with a non-Western migration background. For this purpose, we would like to listen to your practical experiences in order to learn more about this topic. I would like to stress that in this focus group we are not looking for what is right or wrong in dealing with patients and their preferences. However, we are curious to hear how you experience medical decision-making with this diverse patient group, and what exactly you think makes making a decision sometimes challenging or difficult. So thank you all for being here, we are very grateful for your input.
  - Ultimately, a selection of the cases covered in the focus groups will be used later in our research project to conduct further questioning of doctors, patients and lawyers.
- Finally, I would also like to share some practical info:
  - Tonight I will moderate the discussion, that is, I will lead the discussion. Nina and Josefien are both there to help me tonight and can ask you a question from time to time. With that, Nina is mainly the observer tonight, observing the flow of the conversation and making sure everyone gets to speak and that all questions are asked. Josefien/Katrien is my promotor and, as a GP, she has a little more insight into the daily practice of GPs. She will also deal with the technical side tonight, so should anything go wrong with your connection she can help.
  - As agreed beforehand, the conversation will be recorded. That way we can focus on the course of the conversation today, and we don't have to take notes. You will see that we might write something down now and then, but that is more of a reminder for ourselves to ask a certain question.
  - The interaction between you tonight is what will make this conversation interesting. So don't hesitate to pick up on each other's answers, but since we are in an online conversation, it is useful to raise your virtual hand before you speak. Then you'll be let speak as soon as the previous person has finished talking. Besides, it is also better for sound quality to turn off your microphone when you are not speaking.
- Then we can almost start. One last important note is that the things you tell us today will be anonymised. And we would also like to ask you to respect each other's privacy, so who tells what stays among us. Tonight's recording will be kept in a safe place and can only be accessed by the researchers working on this project.
- OK, are there any questions before we get started? Also, anyone know how to raise your hand in Teams? If everything is clear, I will now start the recording.

**Core:**

- Introductory question (everyone (incl ourselves) must answer it): Before we dive into the topic, it's nice to know who we are going to talk to. Could you all introduce yourselves, telling us what practice you are in, what kind of practice it is and where it is located?
- Opening question: To open our conversation on the topic of medical decision-making, I would like to let you brainstorm for a few minutes. After all, as a doctor, during consultations you are constantly making decisions about (and sometimes together with) the patient sitting in front of you. Therefore, we would like to ask you to share a few examples in the chat of such decisions. For example, what decisions have you had to make during a consultation today (or in the past few days)? These can be both easy and difficult decisions. You may share one or a few examples in the chat.
- Tonight we are going to talk about such decisions, but specifically when the patient sitting in front of you has a non-Western migration background. We had given you all 'homework' in advance. We had asked you to think about an experience with a patient from a non-Western migration background, where making a medical decision was challenging.
  - We would love to get everyone talking to listen to your experiences. But maybe there are some of you who already have a very concrete idea in mind to share right now? Because if so, I suggest we start with that. The others can then pick up on this. There is definitely time to discuss multiple experiences tonight.
  - For those participants who already have a concrete idea: Can you raise your hand? I suggest you each already briefly tell the topic of your experience in 1 sentence.
- *Side questions (probes) about the experience:*
  - *What was the patient's presenting complaint?*
  - *What was your working diagnosis?*
  - *Who was present at the consultation (other than yourself and the patient)?*
  - *What was to be decided? (diagnostics, treatment,...)*
  - *What were the patient's preferences (cave: according to the doctor)*
  - *Which community did this patient belong to, and do you know how much of a migrant generation?*
  - *Can you try describing what you found difficult about this case?*
  - *You mention ... . Exactly how does that play a role in making a decision?*
  - *If difficult to say: 'What aspects in that situation made this challenging? Did it have to do with communicating information? Or who exactly was involved in the decision?"*
  - *With each experience, reflection, it is interesting to hear: Is this recognisable to anyone else? Has anyone experienced something similar? What was the context in that situation (share and compare)?*
  - *We will have each participant narrate one case.*
- Patients may want to involve their families to a greater or lesser extent in determining the care they receive. Do you sometimes make decisions with the family?
  - Can you share an experience of a case (in which this was difficult)?
  - What made this a difficult process?
  - Suppose this has already been addressed:
    - In your experiences, it has already come up a few times that having the family closely involved can be challenging. We saw this reflected in decisions about ... but also....
    - Challenging in this is that ... (listing factors). Do I understand so well what you experience in practice?
  - When decisions are made with the family, are there certain situations (e.g. certain themes or disease states) in which this is particularly challenging?
  - When you hear: making decisions together with the family, what do you spontaneously think of? Are these concrete experiences?
- For a patient to participate in decision-making, you have to inform them first. Do you have experiences where communicating medical information with the patient was difficult?
  - What made this difficult?
  - Suppose this has already been addressed:
    - Your experiences have also touched on a few times that communicating information can be challenging. We saw this reflected in decisions about... but also....
    - What played a role in this according to you guys was ... (list factors). Do I understand so well what you experience in practice?
  - When it comes to communicating medical information, are there certain situations (e.g. certain topics or clinical pictures) in which this is particularly challenging?
  - Are there any experiences that come to mind when it comes to communicating information to the patient?
- *Once everyone has shared their experience, it might be time to think about the dimensions we had listed ourselves. For example, the following prompts could be used:*
  - We have heard some experiences about decision-making in physical complaints. Can we reflect together on challenges that may arise when the patient comes with mental complaints and decisions need to be made about them?
  - Your experiences included decisions that had to be made in a (fairly) acute situation. What about decisions about chronic conditions? Does that change anything about the decision-making process?
    - Have you experienced any difficulties in such cases? Can you describe them?
  - The decisions we talked about were often very weighty, with far-reaching consequences. In daily practice, you might also encounter more everyday problems. Do you encounter challenges then?
    - What did you find difficult in that consultation when making a decision?

**Ending:**

- We are now approaching the end of the focus group. Thank you all for sharing your experiences.
- In case our own cases had not yet been covered: We ourselves have already conducted a literature review in preparation for the focus groups. Its results pointed to three more cases that often involve challenging MDM. and we would like to present them to you.
  - Cases involving acute psychiatric problems
    - To what extent can you identify with such a case? Have you experienced any challenges yourselves, specifically with these patient groups?
  - Terminal care
    - To what extent can you identify with such a case study? Have you already experienced challenges yourselves, specifically with these patient groups?
  - Care for chronic diseases: e.g. diabetes that is poorly controlled
    - To what extent can you identify with such a case study? Have you already experienced challenges yourselves, specifically with these patient groups?
- *Overview of the cases cited in the focus group, and what caused challenges in them.* In your opinion, are there any cases or elements/aspects that were not addressed but could still fit into the list?
- Finally, it might also be interesting to talk briefly about the following: because we have talked a lot about the challenges, but do you think there are certain themes or clinical pictures where decision-making goes very smoothly?
- We have now come to the end of the focus group. We would like to thank you all for making time for this, we are very happy with your input. If you have any questions afterwards, you can reach me via e-mail.
- Have a nice evening everyone!

### Focus group discussion with patients: interview guide

**Preparatory Questions (sent in advance via email)**

1. Have you or someone in your environment ever experienced a situation where making a decision about your health was challenging? If so, could you describe this situation?
2. Have you or someone in your community ever felt that the medical care provided by your general practitioner didn’t fully align with your cultural preferences?

**Introduction**

- We are [name(s), background, e.g., sociologist, general practitioner-researcher], and we are collaborating on the first comprehensive, interdisciplinary research project about medical decision-making for patients with a migration background in a European context.
- Medical decision-making refers to all decisions regarding your health that you make during a consultation with your general practitioner. In this study, we are focusing specifically on the Moroccan, Turkish, and Sub-Saharan African communities in Belgium. We’ve chosen these three groups because they are the largest non-Western migrant communities in our country.
- The ultimate goal of this research is to develop recommendations and tools to support general practitioners and other healthcare providers in culturally sensitive medical decision-making. In essence, we aim to create resources that help address the diverse cultural preferences of patients, thereby facilitating healthcare providers’ work and respecting patients’ rights to culturally appropriate care.
- We believe it’s extremely important to involve patients and patient representatives, like you, throughout the research process. This focus group discussion is part of the first phase of the project. We aim to identify challenging cases in medical decision-making so that we can work further on these cases during the research project.
- Today, we hope to explore the difficulties patients with a non-Western background encounter during the medical decision-making process and identify the types of cases where such challenges commonly arise. In later phases, these selected cases will be used to conduct further inquiries with doctors, patients, and legal experts.
- We are very grateful for your presence and valuable contributions today.
- It’s important to note that we’re not looking for what is “right” or “wrong” in how doctors handle patients’ preferences. Instead, the main goal of this discussion is to uncover cases where medical decision-making does not proceed smoothly. Think about situations where the decision-making process taught in Belgian medical practice and patients’ rights seems not to align with the personal and cultural preferences of a patient with a migration background. Ultimately, we want to understand which situations and medical decisions might present challenges, specifically for patients with a Turkish, Moroccan, or Sub-Saharan African background.
- Amina will moderate the discussion, meaning she will guide the conversation. Nina/Flore/Veerle will also be present. They will observe the discussion and intervene if necessary to ensure smooth proceedings, such as making sure everyone gets a chance to speak. They might occasionally take notes—not about what you say specifically, but as reminders for us to ensure all topics are covered and everyone is heard. They will also monitor the time.
- As agreed beforehand, the discussion will be recorded so that we can work with the content later. This allows us to focus on the discussion today without taking extensive notes. These recordings will be used exclusively for this research and will be destroyed afterward. It’s crucial that you’re aware of and agree to this in advance.
- Your interactions will make this discussion meaningful and insightful. You don’t need to raise your hand to speak, but please allow the previous person to finish before you take your turn.
- Does everyone understand this? Are there any questions before we begin? If not, I will now start the recording.

**Focus Group Questions**

**Introductory Questions (for everyone to answer):**

- Before we dive into the topic, it’s nice to know who we’re speaking with. Could everyone introduce themselves and explain their role in this discussion?
- Why do you think you were invited to participate in this focus group?
- What comes to mind when you hear the terms “medical decision-making” and “cultural sensitivity” in one sentence?
- We assigned some “homework” in advance. I’m curious about your responses. Let me repeat the questions for clarity:
  - Have you or someone in your environment ever experienced a situation where making a decision about your health was challenging? If so, could you describe it?
  - Have you or someone in your community ever felt that the medical care provided by your general practitioner didn’t fully align with your cultural preferences? In what situation did this conflict occur?

**Challenges during Consultations**

1. How does your cultural background play a role in making decisions about your health? Do you feel there was space for this during consultations?
2. Have you ever felt that the way you were expected to make a medical decision didn’t align with your cultural preferences? Could you elaborate?
3. Have you experienced tensions during a consultation with your general practitioner? Could you describe this experience?
4. Do you think there are cultural elements that could influence medical decisions? What influences are these?

**Sharing Information**

1. If your doctor provides you with information about your health and asks you to make a choice about further tests or treatments, how do you handle it?
2. What kind of information about your health would you like to receive?
3. Are there certain types of medical information you’d prefer not to receive from your doctor? Could you give an example?
4. How do you view the sharing of information between doctor and patient?
5. Do you think tensions can arise when sharing health information?

**Involving Others in Medical Decision-Making**

1. Would you share information about your health with someone in your environment? If so, with whom?
2. Who do you involve in decisions about your health? Do you bring these individuals to consultations, or do you share and discuss the information afterward?
3. Do you think there is a difference in how first-generation patients of Moroccan/Turkish/Sub-Saharan African backgrounds make decisions about their mental and physical health compared to later generations? Can you give examples?

**Dimensions to Explore**

1. Are there specific situations or illnesses where you think making a health decision is particularly difficult?

If additional themes from literature don’t arise, mention:

- Mental health
- Terminal or palliative care
- Chronic illnesses (e.g., diabetes)

**Closing**

Thank you all for your enthusiastic participation and interaction. It was wonderful to see how everyone shared their perspectives. Is there anything that hasn’t been addressed today that you feel is relevant?

1. Countries of origin of participants with a sub-Saharan African migration background were Angola, Burundi, D.R. Congo, Gambia, and Mali. [↑](#footnote-ref-2)
